# AtezoTRIBE: a randomised phase II study of FOLFOXIRI plus bevacizumab alone or in combination with atezolizumab as initial therapy for patients with unresectable metastatic colorectal cancer

**DOI:** 10.1186/s12885-020-07169-6

**Published:** 2020-07-22

**Authors:** Carlotta Antoniotti, Beatrice Borelli, Daniele Rossini, Filippo Pietrantonio, Federica Morano, Lisa Salvatore, Sara Lonardi, Federica Marmorino, Stefano Tamberi, Salvatore Corallo, Giampaolo Tortora, Francesca Bergamo, Di Stefano Brunella, Alessandra Boccaccino, Elisa Grassi, Patrizia Racca, Emiliano Tamburini, Giuseppe Aprile, Roberto Moretto, Luca Boni, Alfredo Falcone, Chiara Cremolini

**Affiliations:** 1 Department of Oncology, University Hospital of Pisa, Pisa, Italy; 2 Department of Translational Research and New Technologies in Medicine, University of Pisa, Pisa, Italy; 3 Oncology and Hemato-Oncology Department, University of Milan, Milano, Italy; 4 Department of Medical Oncology, Fondazione IRCCS Istituto Nazionale dei Tumori, Milano, Italy; 5 Comprehensive Cancer Center, Fondazione Policlinico Universitario Agostino Gemelli IRCCS, Roma, Italy; 6 Università Cattolica del Sacro Cuore, Roma, Italy; 7 Medical Oncology Unit 1, Department of Clinical and Experimental Oncology, Istituto Oncologico Veneto – IRCCS, Padova, Italy; 8 Medical Oncology, Ospedale degli Infermi, Faenza, Italy; 9 SSD ColoRectal Cancer Unit, Department of Oncology, AOU Città della Salute e della Scienza di Torino, Torino, Italy; 10 Department of Oncology and Palliative Care, Cardinale G. Panico, Tricase City Hospital, Tricase, Italy; 11 Department of Oncology, San Bortolo General Hospital, ULSS8 Berica, East District, Vicenza, Italy; 12 Clinical Trials Coordinating Center, Toscano Cancer Institute, University Hospital Careggi, Firenze, Italy

**Keywords:** FOLFOXIRI, Metastatic colorectal cancer, Immunotherapy, Immune checkpoint inhibitors, Atezolizumab, Chemotherapy, Triplet, Bevacizumab, Microsatellite status

## Abstract

**Background:**

Immune checkpoint inhibitors (ICIs) reported remarkable achievements in several solid tumours. However, in metastatic colorectal cancer (mCRC) promising results are limited to patients with deficient mismatch repair/microsatellite instability-high (dMMR/MSI-high) tumours due to their immune-enriched microenvironment. Combining cytotoxic agents and bevacizumab in mCRC with proficient mismatch repair/microsatellite stability (pMMR/MSS) could make ICIs efficacious by increasing the exposure of neoantigens, especially with highly active chemotherapy regimens, inducing immunogenic cell death, increasing the tumoral infiltration of CD8+ T-cells and reducing tumour-associated myeloid-derived suppressor cells. VEGF-blockade also plays an immunomodulatory role by inhibiting the expansion of T regulatory lymphocytes.

Consistently with this rationale, a phase Ib study combined the anti-PDL-1 atezolizumab with FOLFOX/bevacizumab as first-line treatment of mCRC, irrespective of microsatellite status, and reported interesting activity and efficacy results, without safety concerns.

Phase III trials led to identify FOLFOXIRI plus bevacizumab as an upfront therapeutic option in selected mCRC patients. Drawing from these considerations, the combination of atezolizumab with an intensified upfront treatment (FOLFOXIRI) and bevacizumab could be worthy of investigation.

**Methods:**

AtezoTRIBE is a prospective, open label, phase II, comparative trial in which initially unresectable and previously untreated mCRC patients, irrespective of microsatellite status, are randomized in a 1:2 ratio to receive up to 8 cycles of FOLFOXIRI/bevacizumab alone or in combination with atezolizumab, followed by maintenance with bevacizumab plus 5-fluoruracil/leucovorin with or without atezolizumab according to treatment arm until disease progression. The primary endpoint is PFS. Assuming a median PFS of 12 months for standard arm, 201 patients should be randomized in a 1:2 ratio to detect a hazard ratio of 0.66 in favour of the experimental arm. A safety run-in phase including the first 6 patients enrolled in the FOLFOXIRI/bevacizumab/atezolizumab arm was planned, and no unexpected adverse events or severe toxicities were highlighted by the Safety Monitoring Committee.

**Discussion:**

The AtezoTRIBE study aims at assessing whether the addition of atezolizumab to an intensified chemotherapy plus bevacizumab might be an efficacious upfront strategy for the treatment of mCRC, irrespective of the microsatellite status.

**Trial registration:**

AtezoTRIBE is registered at Clinicaltrials.gov (NCT03721653), October 26th, 2018 and at EUDRACT (2017–000977-35), Februray 28th, 2017**.**

## Background

Tailoring the optimal upfront treatment marks a crucial step in the therapeutic pathway of metastatic colorectal cancer (mCRC), being a primary determinant of every patient’s long-term outcome. The upfront treatment aims at achieving disease control, thus enabling further treatments, and providing a chance of cure in selected cases.

Nowadays, combining a chemotherapy backbone with a biological agent is a standard choice, and a growing amount of clinical evidence supports the modulation of the intensity of the first-line chemotherapy from one- to three-drug regimens according to patients’ and disease characteristics in the perspective of treatments’ personalization [1–3].

To this regard, the phase III TRIBE study compared the triplet FOLFOXIRI plus bevacizumab with the doublet FOLFIRI plus bevacizumab in previously untreated mCRC patients, demonstrating a significant benefit from the intensification of the chemotherapy backbone in terms of progression-free survival (PFS) (primary endpoint, 12.3 versus 9.7 months, hazard ratio (HR) 0.77 (95% CI 0.65–0.93), *p* = 0.006), RECIST response rate (65% versus 53%, p = 0.006) and overall survival (OS) (29.8 versus 25.8 months, HR: 0.80 (95% CI 0.65–0.98), *p* = 0.03) [4, 5].

Moreover, the positive impact of an intensified treatment as first-line therapy on the long-term outcome of mCRC patients has been recently corroborated by the results of the phase III randomized TRIBE2 study. The trial showed a significant benefit in the clinical outcome of mCRC patients from the upfront exposure to FOLFOXIRI plus bevacizumab and its re-introduction at the time of disease progression, as compared to a pre-planned sequence of doublets (first-line mFOLFOX6, followed by FOLFIRI after disease progression) plus bevacizumab across two subsequent lines of therapy, consistently in terms of progression-free survival 2 (PFS2) (primary endpoint, 19.2 versus 16.4 months, HR: 0.74 (95% CI 0.63–0.88), *p* < 0.001), 1st line PFS (12.0 versus 9.8 months, HR 0.74 (95% CI 0·63–0·86), p < 0.001), and OS (27.4 versus 22.5 months, HR: 0.82 (95% CI 0.68–0.98), *p* = 0.032) [6].

Based on these results and other phase II randomized trials, FOLFOXIRI plus bevacizumab is now recommended by major guidelines as a valuable option for the upfront treatment of selected patients with mCRC [1–3].

While the introduction of immunotherapy with Programmed Death 1, Programmed Death-Ligand 1 (PD-1 or PD-L1) or Cytotoxic T-Lymphocyte Antigen 4 (CTLA-4) immune checkpoint inhibitors (ICIs) has deeply changed the treatment algorithm of different tumour types [7–11], the benefit from ICIs is restricted to the small subset of mCRC patients, approximately 3–5% of cases, with microsatellite instability-high (MSI-high) or deficient mismatch repair (dMMR) tumours.

Indeed, interesting results were firstly reported with the anti-PD-1 pembrolizumab in a small cohort of chemorefractory patients. The immune-related objective response rate (irORR) and 20 week-immune-related progression-free rate were 40 and 78%, respectively, for patients with dMMR/MSI-high mCRC, and 0 and 11%, respectively, for those with mismatch repair proficient (pMMR) or microsatellite stable (MSS) disease, leading to hypothesize that mismatch repair (MMR) status could predict the benefit from immunotherapy [12]. The efficacy, together with a manageable safety profile, of pembrolizumab in pretreated patients with dMMR/MSI-high mCRC has been confirmed by the results of the phase II Keynote-164 study [13].

More recently, the clinical benefit of PD-1 blockade alone or in combination with CTLA-4 inhibition in previously treated dMMR/MSI-high mCRC has also been reported in the phase II non comparative CheckMate-142 study. Nivolumab monotherapy or in combination with low-dose ipilimumab provided high response rates (ORR 31 and 55%, respectively), relevant PFS and OS durations, and manageable safety profiles [14, 15]. Furthermore, the combination of nivolumab and ipilimumab confirmed robust and durable clinical benefit, in terms of ORR and 12-months PFS rate (60 and 77%, respectively), and was well tolerated also in patients with previously untreated dMMR/MSI-high mCRC [16].

This clear differential sensitivity to immune checkpoint inhibitors between dMMR/MSI-high and pMMR/MSS mCRC is reasonably due to the different characteristics of the microenvironment in these tumour types. While dMMR/MSI-high tumours have a more active immune background, due to a high burden of neoantigens arising from the hypermutated state of the tumour cells, able to trigger a potent immune response, pMMR/MSS tumours are mostly immune excluded or immune desert, because of a poor or absent T-cell infiltration and a reduced expression of checkpoint proteins [17].

Moving from this biological rationale, strategies potentially able to recruit activated immune cells in the tumour microenvironment, to increase major histocompatibility complex class 1 (MHC-1) and checkpoint proteins expression on tumour cells, to downregulate multiple immunosuppressive cytokines and receptors, thus leading to durable anti-tumour immunity, may be able to overcome ICIs resistance in pMMR/MSS mCRC tumours. To this purpose, several research groups investigated the association of immunotherapy to other drugs with proven immunomodulatory properties, such as chemotherapy, VEGF or MEK inhibitors, in order to sensitize to ICIs otherwise resistant pMMR/MSS tumours.

The phase III IMblaze370 trial assessed the efficacy of atezolizumab, a PD-L1 inhibitor, either plus cobimetinib, a MEK-1 and -2 inhibitor, or alone, versus regorafenib in a cohort of chemorefractory mCRC patients. The enrolment of those with dMMR/MSI-high disease was restricted to the 5% of the whole study population. The trial failed to demonstrate an improvement in OS, the primary endpoint, with atezolizumab plus cobimetinib or atezolizumab alone versus regorafenib [18].

In the first-line induction setting, encouraging results were reported in a phase Ib study of FOLFOX plus bevacizumab and atezolizumab, showing interesting activity (ORR: 48%) and a reassuring safety profile, with no unexpected adverse events or exacerbation of chemotherapy- or bevacizumab-related toxicities [19]. From a biological perspective, by comparing pre- and post-treatment tumour tissues, combining FOLFOX plus bevacizumab and atezolizumab significantly increases CD8+ T cells and PD-L1 expression, as compared to FOLFOX alone. These modifications seem to promote immune-related activity and potentially result in enhanced efficacy [20].

**Fig. 1:**
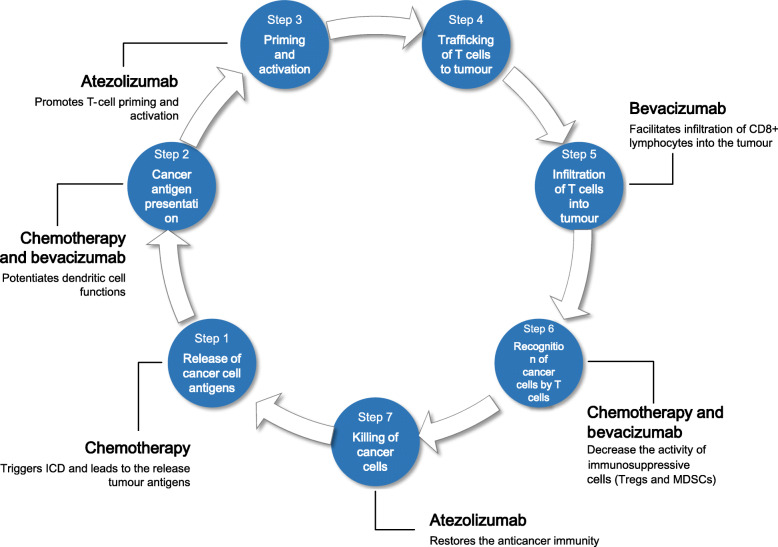
Rationale for combining chemotherapy, bevazicumab and atezolizumab. DC, dendritic cell; ICD, immunogenic cell death; MDSCs, myeloid-derived suppressor cells; Tregs, regulatory T cells; VEGF: Vascular Endothelial Growth Factor

These results strengthen the hypothesis that combining chemotherapy and bevacizumab could definitely modify the tumour immune microenvironment, by working on multiple steps in the anti-cancer immunity process (Fig. 1). Taken together, these effects could synergically make ICI-based strategies more effective in activating the local immune system against cancer cells.

In particular, the immunogenicity of active chemotherapy regimens is due to the ability to induce cell death and to activate T cells as consequence of the resultant antigens’ release, thus tipping the balance between effector and regulatory/suppressor cells in favour of the firsts. In more details, looking at the cytotoxic drugs commonly used in the treatment of mCRC, 5-fluoruracil (5FU) has a strong ability in selectively depleting tumour-associated myeloid-derived suppressor cells (MDSCs) and in increasing CD8+ tumour-infiltrating lymphocytes [21], while oxaliplatin potentiates dendritic cell (DC) functions and induces immunogenic cell death (ICD) and irinotecan inhibits the immunosuppressive environment [22, 23].

On the other hand, the vascular endothelial growth factor (VEGF) is a proangiogenic factor with immunomodulatory effects. Indeed, VEGF directly suppresses various immune cells present in the tumour microenvironment, contributes to tumour associated immune deficiency, inhibits the antigen presentation process by DCs; induces apoptotic death in CD8+ T cells and promotes the activity of T regulatory cells (Tregs).

Therefore, by blocking VEGF-VEGFR pathway, bevacizumab is able to restore the immune adaptive mechanisms of the tumour microenvironment, enhancing T-cell priming and activation via promotion of DCs maturation, increasing effector T-cells tumour infiltration by normalising tumour vasculature, and establishing an immune-permissive tumour microenvironment by reducing level of Treg and tumour-associated MDSC populations [24–27]. As a consequence, through the reversal of VEGF-mediated immunosuppressive effects, VEGF blockade by bevacizumab could amplify T-cell-mediated cancer-cell killing by ICIs. Finally, it is well established the role of atezolizumab in restoring T-cell activity against cancer cells through the inhibition of PD-L1 on tumour cells surface [28].

Moving from these considerations, we designed the AtezoTRIBE study, a phase II randomised trial conceived with the aim of verifying whether the addition of the anti-PD-L1 atezolizumab to a highly active first-line induction treatment, the triplet FOLFOXIRI plus bevacizumab, could improve the outcome of patients with unresectable mCRC, unselected for the microsatellite status. Herein, we present also the results of the safety run-in phase of the AtezoTRIBE study, planned to early assess the safety and feasibility of the experimental regimen.

## Methods/design

### Study design

AtezoTRIBE is a prospective, open label, phase II, comparative trial in which initially unresectable and previously untreated mCRC patients, irrespectively of the microsatellite status of their tumours, are randomized in a 1:2 ratio to receive induction treatment with FOLFOXIRI plus bevacizumab up to 8 cycles (arm A, standard treatment) or in combination with atezolizumab (arm B, experimental treatment), followed by maintenance with 5-fluoruracil/leucovorin (5FU/LV) plus bevacizumab with or without atezolizumab according to treatment arm until disease progression, unacceptable adverse events or consent withdrawal.

**Fig. 2:**
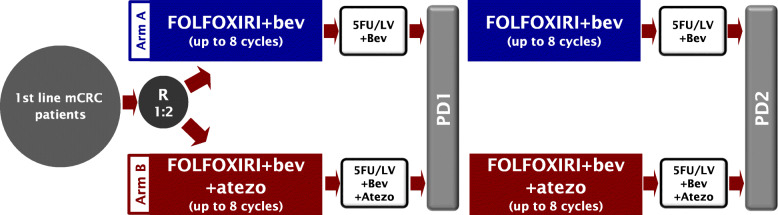
Study design. mCRC, metastatic colorectal cancer; FOLFOXIRI, 5-flurouracil, irinotecan, oxaliplatin; bev, bevacizumab; atezo, atezolizumab; 5FU, 5-fluorouracil; LV, Leucovorin; PD1, first disease progression; PD2 second disease progression

The registration and randomization procedures are centralized at Clinical Trials Coordinating Center - Istituto Toscano Tumori. The randomization is performed by using an electronic WEB-based system according to the minimization algorithm. Stratification criteria are ECOG PS (0 versus 1, 2), primary tumour location (right versus left/rectum) and previous adjuvant chemotherapy (yes versus no). If disease progression does not occur during induction, at the treating physician’s discretion, the reintroduction after progression of the same induction treatment (up to 8 cycles) according to randomization arm, followed by maintenance until disease progression, unacceptable toxicity or patient’s refusal, is recommended (Fig. 2).

A patient may be discontinued from the clinical trial at any time for any reason. It is the right and the duty of the investigator to stop treatment in any case in which emerging effects are of unacceptable risk to the individual subject’s safety, in the best interest of the patient. In addition, patients have the right to voluntarily discontinue study treatment or withdraw from the study at any time for any reason.

The feasibility of surgical radical resection of residual metastases in responsive patients is evaluated every 8 weeks. In the case of secondary resection of metastases, at least 5 weeks should elapse between the last administration of bevacizumab and the day of surgery. A postoperative therapy with the same induction regimen received before resection is planned up to a total duration (preoperative plus postoperative treatment) of 12 cycles, followed by maintenance up to a total of 12 postoperative cycles (including induction and maintenance) according to randomized arm. Post-operative treatment should start not earlier than 4 weeks after surgery.

A safety run-in phase was planned to assess the feasibility of the experimental treatment by observing the first six patients enrolled in arm B. In that phase the study was active only at the Coordinating Centre (Department of Medical Oncology, Azienda Ospedaliero-Universitaria Pisana, Pisa). The enrolment of new patients was then temporary interrupted to allow the Safety Monitoring Committee (SMC) to evaluate the safety of the new combination. Only if the study treatment combination was judged feasible and no major safety concerns arose, the enrolment would be resumed and involved additional participating sites.

### Study objectives and endpoints

The primary objective of this study is to evaluate the efficacy of the addition of atezolizumab to FOLFOXIRI plus bevacizumab as first-line treatment of unresectable mCRC in terms of PFS, defined as the time from randomisation to disease progression or death, whichever occurs first.

Secondary objectives of this trial are to compare the two proposed treatments in terms of safety profile, ORR, immune-related ORR (irORR), early objective overall response rate (EOR) and deepness of response (DoR), as previously defined [29], R0 resection rate, progression free survival 2 (PFS2), 2nd PFS, OS.

ORR and irORR are defined as the percentages of patients, relative to the total of enrolled subjects, achieving a complete (CR) or partial (PR) response, according to RECIST 1.1 criteria [30] and immune-modified RECIST criteria [31], respectively, during the induction and the maintenance phases of treatment. EOR is defined as the percentage of patients, relative to the total of the enrolled subjects, achieving a 20% decrease in the sum of diameters of RECIST target lesions at week 8 compared to baseline; DoR is defined as the relative change in the sum of longest diameters of RECIST target lesions at the nadir, in the absence of new lesions or progression of non-target lesions, when compared with baseline. R0 resection rate is defined as the percentage of patients, relative to the total of enrolled subjects, undergoing secondary R0 resection of metastases; PFS2 is defined as beginning with randomization and ending with death or disease progression according to RECIST 1.1 criteria on any treatment given after first progression; OS is defined as the time from randomisation to the date of death due to any cause.

### Statistical design and sample size calculation

The primary analysis of PFS will be performed in the intention-to-treat population. The Kaplan-Meier approach will be used to estimate survival analysis. Log-rank test stratified by the same factors as used for randomization will also be performed, as well as a multivariable model including all the significant baseline variables. Based on the assumption that PFS of each arm follows an exponential distribution and considering an expected median PFS of 12 months for standard arm, 129 events are required to detect a hazard ratio (HR) for PFS of 0.66 in favour of the experimental group (arm B), with a one-sided unstratified log-rank test, with α and β errors of 0.10 and 0.15, respectively. Assuming an accrual rate of 210 subjects/year, a 1:2 randomization and a minimum follow up period equal to 1.5 years, a total of 201 patients should be randomized (arm A/B: 67/134).

*Post-hoc* exploratory subgroup analyses will be performed with an interaction test to assess the consistency of the treatment effect according to key baseline characteristics, including the microsatellite status.

### Study population

The study has been approved by 25 ethics committees and is currently ongoing at 25 Italian oncology units. Main inclusion criteria are: mCRC patients with Eastern Cooperative Oncology Group (ECOG) Performance Status (PS) ≤2 if aged < 70 years, or ECOG PS 0 if aged 71–75 years; the availability of tumour tissue samples (primary and/or metastatic sites), at least one measurable lesion according to RECIST 1.1 criteria, adequate liver, renal and bone marrow function. Main exclusion criteria are: oxaliplatin-based adjuvant chemotherapy and history of autoimmune disease. Adjuvant treatment with fluoropyrimidine alone is allowed if relapse occurs after more than 6 months from the end of therapy.

### Study procedures and safety

Eligible patients are randomized to receive FOLFOXIRI plus bevacizumab (arm A, bevacizumab 5 mg/kg, irinotecan 165 mg/m^2^, L-leucovorin (LV) 200 mg/m^2^, oxaliplatin 85 mg/m^2^, 5-fluorouracil 3200 mg/m^2^ 48-h continuous infusion) every 2 weeks for a maximum of 8 cycles, or FOLFOXIRI plus bevacizumab at the same doses plus atezolizumab 840 mg every 2 weeks for a maximum of 8 cycles (arm B). Following the induction phase, if no progression occurs, maintenance with 5FU/LV plus bevacizumab alone or with atezolizumab, according to the randomization arm, is administered biweekly in both arms at the same dose used at the last cycle of the induction treatment until disease progression, unacceptable toxicity or patient’s refusal.

Tumour assessment is performed by means of CT scan every 8 weeks, according to RECIST version 1.1 criteria [30].

In order to standardize the use of corticosteroids, the protocol recommends 12 mg of dexamethasone or equivalent and 5-HT_3_ antagonist at day1 within 1 h before and the day after the administration of the study drugs, as antiemetic prophylaxis.

All adverse events observed during the study treatment period are registered in the subjects’ medical records and in the electronic case report forms (ecrfs), according to National Cancer Institute Common Terminology Criteria for Adverse Events (NCI-CTCAE) version 4.0 criteria [32]. Any serious adverse event (SAE) defined as an adverse event which is fatal or life-threatening, requiring hospitalization or resulting in persistent or significant disability/incapacity, and non-serious and serious adverse event of special interest (AESI) attributable to bevacizumab or atezolizumab should be notified by the investigator to the Sponsor within 24 h after learning of the event according to local procedures, statutes and the European Clinical Trial Directive (when applicable). The Sponsor medically reviews all SAEs and AESIs and is responsible for their notification to the appropriate ethics committees, competent authorities and participating Investigators.

Dose reductions and delays are detailed in the study protocol, as well as recommendations about the management of specific adverse events that may be potentially related to different drugs (i.e. diarrhoea). To this purpose, investigators should exploit their knowledge of the patient, the circumstances surrounding the event, the evaluation of any potential alternative causes to determine whether an adverse event is considered related or not to one or more study drugs. Also the course of the event and how it is modified by recommended interventions (i.e., dose reduction, discontinuation or reintroduction of study drug, supportive therapy), may help to distinguish among different potential causes.

### Translational analyses, ethics and regulatory considerations

A program of translational analyses is planned.

The availability of a tumour specimens (primary tumour and/or metastases if available) collected at first diagnosis is mandatorily required for participation in this study. Tissue samples are collected also in the case of secondary surgery with curative intent during study treatment. Exploratory biomarker translational analyses, including but not limited to the evaluation of immunity-related parameters, on tumour samples collected before and after the treatment, will be performed in an effort to understand the association of these markers with study treatment outcome.

Also blood and plasma samples are collected at baseline, at the 2nd cycle, at the end of the induction phase, and at the time of first and second progression. After a recent amendment, the collection of faecal material (at baseline, at the end of the induction phase and at the time of first progression) is planned, in order to study how the composition of intestinal microbiota might modulate the response to chemotherapy plus bevacizumab with or without immunotherapy. A comprehensive genome profiling analysis including the assessment of tumour mutational burden is planned.

All the investigators involved in the present study respect the Good Clinical Practice guidelines and the latest version of the Declaration of Helsinki. All patients provide a written informed consent to study procedures before the enrolment. Investigators are responsible for informing each patient (or legally authorized representative) of the nature of the study, its purpose, the procedures involved, the expected duration, the potential risks and benefits involved and any discomfort it may entail.

The trial was registered in the EUDRACT database (EUDRACT NUMBER 2017–000977-35) on February 28th, 2017 and in the clinicaltrials.gov registry (ClinicalTrials.gov Identifier NCT03721653; https://clinicaltrials.gov/ct2/show/NCT03721653) on October 26th 2018.

### Coordination

Department of Medical Oncology, Azienda Ospedaliero-Universitaria Pisana (Pisa, Italy) is responsible for the overall coordination and management of the study on behalf of GONO Foundation.

## Safety run-in phase results

Between November 30th, 2018 and March 2nd, 2019, a safety run-in phase was conducted at the study Coordinating Center. Eight patients were randomly assigned and received study treatment (2 in arm A and 6 in arm B). According to the study protocol, the SMC reviewed the safety data when 6 patients enrolled in arm B had received at least 2 cycles of the study treatment.

A total of 30 cycles of treatment were administered: 9 cycles of FOLFOXIRI plus bevacizumab in arm A and 21 cycles of FOLFOXIRI plus bevacizumab plus atezolizumab in arm B.

All patients enrolled in this phase were assessed for safety. Maximum grade of adverse events was 3. No grade 4 or Serious Adverse Events were reported. Grade 3 neutropenia was observed in two patients, one patient in arm A and one in arm B, both reasonably related to FOLFOXIRI. One patient in arm A experienced Grade 3 diarrhoea and one patient in arm B experienced Grade 3 hypertension reasonably related to FOLFOXIRI and bevacizumab, respectively. Treatment was delayed because of any adverse event in 3 cycles: one was due to Grade 3 neutropenia in a patient enrolled in arm B after the first cycle, and two were observed in one patient in arm A for Grade 3 neutropenia at the beginning of the second cycle and for Grade 3 diarrhoea at the fifth cycle, respectively. One dose reduction of 5-fluorouracil and irinotecan (both at 75% of the planned dose) was required for one patient in arm A for Grade 3 diarrhoea experienced after the fourth cycle of treatment. No major safety concerns or unexpected adverse events were observed. As a consequence, the SMC judged the study combination well tolerated and feasible and worthy of further investigation. The enrolment restarted and is currently ongoing at 25 Italian participating sites.

## Discussion

To date, the combination of FOLFOXIRI plus bevacizumab is a standard therapeutic option for the upfront treatment of selected patients with mCRC [1–3].

Recently new data in favour of the upfront treatment with triplet plus bevacizumab in mCRC were reported by the TRIBE2 study, which aimed at answering some open questions that partially limited the adoption of FOLFOXIRI plus bevacizumab in the daily practice. Indeed, TRIBE2 study clearly demonstrated the positive impact of the use of FOLFOXIRI plus bevacizumab, administered for a short induction period (i.e., up to 8 cycles), on the long-term outcome of patients with unresectable mCRC, showing the superiority of this upfront intensified strategy, also when compared with the sequential exposure to doublets plus bevacizumab. Furthermore, when choosing the upfront exposure to the three cytotoxics, the feasibility and the efficacy of treatments given after disease progression were not impaired, and the re-induction with the triplet could provide a further benefit in delaying tumour progression [6].

These results strengthened the design of the present study, where combination regimens are restricted up to 8 cycles followed by a bevacizumab- and 5FU/LV-based maintenance, alone or in combination with atezolizumab according to the randomization arm, until disease progression in both arms. At the first evidence of disease progression, the re-introduction of the treatment received upfront is planned, when clinically feasible.

In order to overcome the primary resistance to immunotherapy of pMMR/MSS mCRCs, many attempts tried to test novel therapeutic combinations that could potentially make pMMR/MSS mCRC “immune-competent” and therefore amenable for effective immunotherapy-based strategies.

Among studies addressing this challenge in the early phases of metastatic disease, unsatisfactory results about the combination of immune checkpoint inhibitors with chemotherapy and a biologic agent were reported in the setting of maintenance following a first-line therapy in the MODUL trial [33]. In this randomized umbrella study, after receiving an induction with FOLFOX plus bevacizumab, patients were treated with maintenance with fluoropyrimidine plus bevacizumab alone (control arm) or with an experimental biomarker-driven treatment (cohort 1, for patients with *BRAF* mutant disease: 5FU/LV plus cetuximab and vemurafenib; cohort 2, for patients with *BRAF* wild-type disease: fluoropyrimidine plus bevacizumab and atezolizumab; cohort 3, for patients with *HER2*-positive disease: capecitabine plus trastuzumab and pertuzumab; cohort 4, for patients with *HER2*-negative and *BRAF* wild-type disease: cobimetinib plus atezolizumab). Data from the cohort 2, in which patients were treated with maintenance with fluoropyrimidine/bevacizumab with or without atezolizumab, showed that there was no improvement in progression-free survival, the primary endpoint, from the addition of atezolizumab to a standard first-line maintenance regimen.

Similarly, the phase II randomized BACCI study demonstrated a weak signal of efficacy in delaying tumour progression in the refractory setting, when adding atezolizumab to capecitabine and bevacizumab in pre-treated patients with mCRC unselected for the microsatellite status [34].

These disappointing findings may be partially explained by the limited ability of a fluoropyrimidine alone, in combination with bevacizumab, to augment the immunogenicity of tumour cells and, therefore, to favour the anti-tumour activity of atezolizumab. In fact, it is conceivable that a more active chemotherapy combination might have a more pronounced direct cytotoxic effect on cancer cells, by inducing increased immunogenic cell death and release of tumour-associated neoantigens, thus leading to a more effective stimulation of the host immune response. Consequently, the de-intensified chemo-backbone adopted in the setting of maintenance therapy may be suboptimal to assess the added value of combining an immune checkpoint inhibitor.

According to these working hypotheses, we designed the AtezoTRIBE study with the aim of evaluating whether the addition of atezolizumab to upfront FOLFOXIRI plus bevacizumab might be beneficial in the first-line setting. In particular, the high activity of the triplet FOLFOXIRI may target the poor antigenicity of pMMR/MSS colorectal cancer by enhancing the immunogenic cell death, while bevacizumab counteracts mechanisms of immune-tolerance.

The planned program of translational analyses, based on the collection tumour tissues, blood, plasma and faecal samples, at baseline and during the study treatment, will take advantage of the randomized design of the study. This is a unique chance to provide a comprehensive characterization of immunologic evolutionary dynamics occurring as a consequence of the pressure exerted by each tested treatment in a well-annotated series of patients included in a controlled clinical trial. Findings about whether and how an intensive triple chemotherapy regimen plus bevacizumab alone or in combination with an immune checkpoint inhibitor, such as atezolizumab, could affect and shape the immune background of the tumour and the host may be hypothesis-generating and have major implications in conceiving new therapeutic approaches by better exploiting the use of immunotherapy.

## Supplementary information

**Additional file 1.** List of Ethics Committees that approved the study protocol.

## Data Availability

Data sharing is not applicable to this article.

## Abbreviations

ICIs: immune checkpoint inhibitors
mCRC: metastatic colorectal cancer
5FU: 5-fluorouracil
LV: leucovorin
GONO: Gruppo Oncologico del Nord-Ovest
PFS: progression-free survival
OS: overall survival
PFS2: progression-free survival 2
PD-1: programmed death 1
PD-L1: programmed death-ligand 1
CTLA-4: cytotoxic T-Lymphocyte Antigen 4
MSI-high: microsatellite instability-high
dMMR: deficient mismatch repair
irORR: immune-related objective response rate
pMMR: proficient mismatch repair
ORR: objective response rate
MHC-1: major histocompatibility complex class 1
VEGF: vascular endothelial growth factor
MEK: mitogen-activated protein kinase enzyme
MDSC: myeloid-derived suppressor cell
DC: dendritic cell
ICD: immunogenic cell death
Tregs: T-regulatory cells
ECOG PS: Eastern Cooperative Oncology Group Performance Status
SMC: Safety Monitoring Committee
EOR: early objective overall response rate
DoR: deepness of response
CR: complete response
PR: partial response
HR: hazard ratio
RECIST: Response Evaluation Criteria in Solid Tumours
NCI-CTCAE: National Cancer Institute Common Terminology Criteria for Adverse Events
SAE: serious adverse event
AESI: adverse event of special interest
HER2: human epidermal growth factor receptor 2
