# Supplementary material for: AtezoTRIBE: a randomised phase II study of FOLFOXIRI plus bevacizumab alone or in combination with atezolizumab as initial therapy for patients with unresectable metastatic colorectal cancer
Source: BMC Cancer. 2020 Jul 22;20:683. doi: 10.1186/s12885-020-07169-6 (PMC7376656; doi:10.1186/s12885-020-07169-6)
Supplement: Supplementary file 1 — Additional file 1. List of Ethics Committees that approved the study protocol. [file 12885_2020_7169_MOESM1_ESM.docx]

**Additional file 1. List of Ethics Committees that approved the study protocol**

| **CITY** | **SITE NAME** | **EC name** | **STUDY REFERENCE NUMBER** |
| --- | --- | --- | --- |
| Pisa | Azienda Ospedaliero Univeristaria Pisana | Comitato Etico Area Vasta Nord Ovest  Sezione Autonoma Del Comitato Etico Regionale   per la Sperimentazione Clinica | 13582_Cremolini |
| Aviano | Centro di riferimento Oncologico di Aviano | Comitato Etico Unico Regionale | CEUR 2019  sper 148 |
| Brescia | Fondazione Poliambulanza Istituto Ospedaliero | Comitato Etico Provinciale della Provincia di Brescia | NP 2955 |
| Catania | ARNAS GARIBALDI, P.O. Nesima | Comitato Etico Catania 2 | 54/2019/CETC2 |
| Cremona | Istituti Ospitalieri di Cremona | Comitato Etico Area Cremona, Mantova e Lodi | 16-2019-SPERIM_ FARM-CR10 |
| Cuneo | Azienda Sanitaria Ospedaliera Santa Croce e Carle | Comitato Etico Interaziendale dell’ASO S. Croce e Carle di Cuneo e delle AA.SS.LL. CN1, CN2 e ASTI | 56-2019 |
| Firenze | Azienda Ospedaliero - Universitaria Careggi | Comitato Etico Area Vasta Centro | 12089_spe |
| Frosinone | Polo Oncologico Provinciale Frosinone Azienda Sanitaria Locale | Comitato Etico Lazio 2 | 02.19 |
| Livorno | Ospedali Riuniti di Livorno | Comitato Etico Area Vasta Nord Ovest  Sezione Autonoma Del Comitato Etico Regionale   per la Sperimentazione Clinica | 13582_Livorno |
| Meldola | IRCCS – IRST, Istituto Scientifico Romagnolo per lo Studio e la Cura dei Tumori (I.R.S.T.) | COMITATO ETICO ROMAGNA | 2252 |
| Milano | Istituto Nazionale dei Tumori | Comitato Etico della Fondazione IRCCS “Istituto Nazionale Tumori” | INT 139/18 |
| Modena | Azienda Ospedaliero Universitaria Policlinico di Modena | Comitato Etico dell’Area Vasta Emilia Nord | 129/2019/FARM/AOUMO |
| Napoli | Azienda Ospedaliera Seconda Università Degli Studi di Napoli (SUN) | Comitato Etico della Seconda Università di Napoli | 231/2018 |
| Padova | Istituto Oncologico Veneto | Comitato Etico per la Sperimentazione Clinica (CESC) | 2018/01/RIP |
| Pontedera | Ospedale Felice Lotti Pontedera | Comitato Etico Area Vasta Nord Ovest  Sezione Autonoma Del Comitato Etico Regionale   per la Sperimentazione Clinica | 13582_Pontedera |
| Prato | Nuovo Ospedale di Prato | Comitato Etico Area Vasta Centro | 12089_spe |
| Faenza | A.USL Romagna | COMITATO ETICO ROMAGNA | 2252 |
| Roma | Ospedale San Giovanni Calibita Fatebenefratelli Isola Tiberina | Comitato Etico Lazio 1 | 638 |
| Roma | Policlinico Unversitario Campus Bio-Medico | Comitato Etico Università Campus Bio Medico di Roma | 21.19 |
| Siena | Azienda USL Toscana-Sud Est,Ospedale Alta Valdelsa | Comitato Etico Area Vasta Sud Est | CE 13582 |
| Tricase | Azienda Ospedaliera "Card. G. Panico" | COMITATO ETICO DELL'ASL DI LECCE | 2019/3633 |
| Torino | Azienda Ospedaliero - Universitaria San Giovanni Battista di Torino | Comitato Etico Interaziendale A.O.U. Città della Salute e della Scienza di Torino – A.O. ordine Mauriziano - A.S.L. TO1 | CS2/1185 |
| Udine | A.O. Universitaria Santa Maria della Misericordia | Comitato Etico Unico Regionale | CEUR 2018 sper 135 ASUIUD |
| Roma | Policlinico Gemelli | Fondazione Policlinico Universitario Gemelli Università Cattolica del sacro cuore | 50784/18 ID2334 |
| Vicenza | Azienda ULSS 6 "Vicenza" | Comitato Etico per le sperimentazioni cliniche della provincia di Vicenza | 102/18 |
